# Maldistribution of pulmonary blood flow in patients after the Fontan operation is associated with worse exercise capacity

**DOI:** 10.1186/s12968-018-0505-4

**Published:** 2018-12-17

**Authors:** Tarek Alsaied, Lynn A. Sleeper, Marco Masci, Sunil J. Ghelani, Nina Azcue, Tal Geva, Andrew J. Powell, Rahul H. Rathod

**Affiliations:** 1 Department of Cardiology, Boston Children’s Hospital, Boston, MA USA; 2 Department of Pediatrics, Harvard Medical School, Boston, MA USA

**Keywords:** Fontan procedure, Maldistribution of pulmonary blood flow, Cardiovascular magnetic resonance imaging, Congenital heart disease, Exercise capacity

## Abstract

**Background:**

Maldistribution of pulmonary artery blood flow (MPBF) is a potential complication in patients who have undergone single ventricle palliation culminating in the Fontan procedure. Cardiovascular magnetic resonance (CMR) is the best modality that can evaluate MPBF in this population. The purpose of this study is to identify the prevalence and associations of MPBF and to determine the impact of MPBF on exercise capacity after the Fontan operation.

**Methods:**

This retrospective single-center study included all patients after Fontan operation who had maximal cardiopulmonary exercise test (CPET) and CMR with flow measurements of the branch pulmonary arteries. MPBF was defined as > 20% difference in branch pulmonary artery flow. Exercise capacity was measured as percent of predicted oxygen consumption at peak exercise (% predicted VO_2_). Linear and logistic regression models were used to determine univariate and multivariable predictors of exercise capacity and correlates of MPBF, respectively.

**Results:**

A total of 147 patients who had CMR between 1999 and 2017 were included (median age at CMR 21.8 years [interquartile range (IQR) 16.5–30.6]) and the median time between CMR and CPET was 2.8 months [IQR 0–13.8]. Fifty-three patients (36%) had MPBF (95% CI 29–45%). The mean % predicted VO_2_ was 63 ± 16%. Patients with MPBF had lower mean % predicted VO_2_ compared to patients without MPBF (60 ± 14% versus 65 ± 16%, *p* = 0.04). On multivariable analysis, a lower % predicted VO_2_ was independently associated with longer time since Fontan, higher ventricular mass-to-volume ratio, and MPBF. On multivariable analysis, only compression of the branch pulmonary arteries by the ascending aorta or aortic root was associated with MPBF (OR 6.5, 95% CI 5.6–7.4, *p* < 0.001).

**Conclusion:**

In patients after the Fontan operation, MPBF is common and is independently associated with lower exercise capacity. MPBF was most likely to be caused by pulmonary artery compression by the aortic root or the ascending aorta. This study identifies MPBF as an important risk factor and as a potential target for therapeutic interventions in this fragile patient population.

## Background

Despite the significant improvement in outcomes after the Fontan operation, complications and comorbidities are still common [1, 2]. Optimizing the Fontan circuit is an important factor in reducing these comorbidities [3]. Cardiovascular magnetic resonance (CMR) has been shown to be a valuable tool to predict adverse outcomes in Fontan patients. One of the quantitative measurements by CMR is blood flow to each branch pulmonary artery [4, 5]. Studies evaluating Fontan patients have suggested that up to 45% of patients have maldistribution of pulmonary blood flow (MPBF), defined as a difference between left pulmonary artery (LPA) and right pulmonary artery (RPA) blood flow of > 20% [4].

The etiology of MPBF in the Fontan circulation is likely multifactorial due to different anatomic and physiologic abnormalities [6]. Some studies suggest that the LPA can become compressed by the aortic root or ascending aorta, especially in patients with hypoplastic left heart syndrome (HLHS) [7]. RPA twisting may be associated with the extracardiac Fontan modification [8]. Lung pathologies including pulmonary hypoplasia and pulmonary vascular disease are common in Fontan physiology and may also result in MPBF [6]. Furthermore, compression of pulmonary venous return may occur due to atrial dilation or by the Fontan conduit or baffle [9, 10]. These subtle abnormalities can lead to MPBF which may result in adverse hemodynamics. The associations of MPBF with exercise capacity and other clinical outcomes are largely unknown [11, 12]. The purpose of this study is to identify the impact of MPBF on exercise capacity and clinical outcomes in patients after the Fontan operation.

## Methods

### Patients

A database search identified all post-operative Fontan patients who had a CMR study and cardiopulmonary exercise test (CPET) at Boston Children’s Hospital between January 1999 and July 2017. Patients were included if differential branch pulmonary artery (PA) flow could be calculated by CMR and if they had a maximal effort on exercise stress testing, defined as a respiratory exchange ratio of ≥1.09 or a heart rate of ≥75% predicted. Patients were excluded if there were any interventions or procedures between the CMR and CPET or if the period between CPET and CMR was more than 2 years. The Boston Children’s Hospital Committee on Clinical Investigation approved this retrospective study and waived the requirement for informed consent.

### CMR

CMR studies were performed with 1.5 Tesla scanners (Philips Healthcare, Best, the Netherlands or GE Medical Systems, Milwaukee, Wisconsin). The details of the CMR protocols used in our laboratory for assessment of patients after the Fontan operation have been published [13–15]. Briefly, ventricular assessment was performed by an electrocardiographically-gated, balanced steady-state free precession (bSSFP) cine CMR in vertical and horizontal ventricular long-axis planes, and a stack of slices in a ventricular short-axis plane encompassing the atrioventricular junction through the cardiac apex. Retrospectively cardiac gated, free-breathing, through-plane phase-contrast flow meaurements were obtained in the branch pulmonary arteries and vena cavae. Care was taken to align the imaging plane perpendicular to flow and to obtain slice positions and orientations that were proximal to the PA branching [4].

### CMR data analysis

**Fig. 1:**
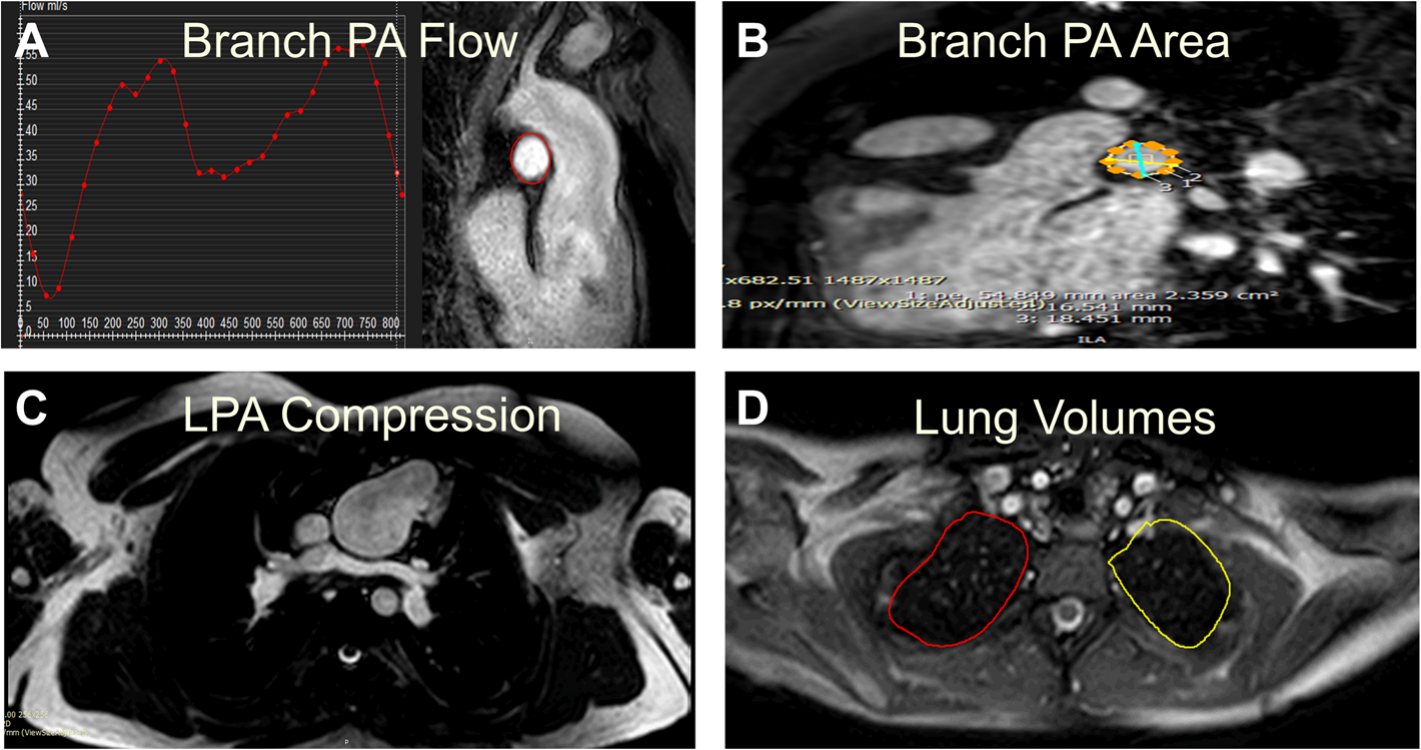
Cardiovascular magnetic resonance (CMR) example of different measurements used in our study. **a** Branch pulmonary artery (PA) flow measurement. **b** Branch PA cross-sectional area was calculated by measuring two orthogonal dimensions at the narrowest segment. **c** Left pulmonary artery (LPA) compression by a dilated ascending aorta. **d** Lung volume calculation by Simpson’s method using manual tracing in each slice on an axial image balanced steady-state free precession stack

If a patient had multiple CMR studies, the most recent study with complete flow data was used for analysis. MPBF was calculated by direct measurement of the branch PA flow on phase contrast imaging. Branch PA flow was measured by manually tracing each branch PA on phase contrast imaging using QFlow (Medis Medical Imaging Systems, Leiden, The Netherlands) (Fig. 1a) [16]. Percentage flow to each PA was calculated. In patients without baffle leaks or patent fenestrations who had unilateral PA stents where direct PA flow could not be measured, flow in the stented PA was calculated using the following formula: superior vena cava flow+ inferior vena cava flow – the non-stented branch PA flow. Branch PA cross-sectional area was calculated by measuring two orthogonal dimensions at the narrowest segment and indexed to body surface area (Fig. 1b). A branch PA symmetry index (PASI) was calculated as the ratio of the area of the smaller pulmonary artery to the larger pulmonary artery. PASI is always ≤1 with values closer to 1 reflecting more symmetric branch PAs [17]. PA compression by the ascending aorta or aortic root was determined by review of CMR images by a provider who was blinded to the PA blood flow distribution. PA compression was defined as narrowing of the branch PA to < 75% of its original diameter as it crossed posterior to the ascending aorta or the aortic root (Fig. 1c) [18]. Lung volumes were calculated by Simpson’s method using manual tracing of the lung fields in each slice on an axial image bSSFP stack (Fig. 1d). Lung volume discrepancy was defined as the absolute value of the difference between the right and left lung volume percentage. Aortic root total area was measured by adding dominant to non-dominant aortic root area as measured on axial planes [19].

Ventricular volumes and function were measured by manual tracing of endocardial and epicardial borders on each short-axis bSSFP cine slice at end-diastole (maximal volume) and end-systole (minimal volume) as previously described [5, 15]. Analysis was performed using commercially available software (QMass, Medis Medical Imaging Systems, Leiden, The Netherlands) and (cmr^42^, Circle Cardiovascular Imaging Inc., Calgary, Canada).

### Clinical parameters

Demographic and clinical data, including underlying diagnoses and type of single ventricle based on ventricular dominance, were abstracted from the medical records. The type of surgical palliation was classified as lateral tunnel, extracardiac conduit, right atrium-to-PA anastomosis, or right atrium-to-right ventricle connection. Additional parameters included age at Fontan, time from Fontan to CMR, and number and type of surgical and catheterization interventions before and after CMR. Arrhythmia history was compiled by review of Holter monitors, electrocardiograms, electrophysiology catheterizations, and clinic notes. Other relevant clinical variables included a history of heart failure (defined as New York Heart Association class II or greater), protein-losing enteropathy, stroke, thrombus, seizures, liver disease, or pacemaker or defibrillator placement.

### Cardiopulmonary exercise testing

A maximal CPET was performed using a calibrated cycle ergometer and ramp protocol (Corival Load Cycle 400, Lode BV, Groningen, The Netherlands). The test starts with setting an initial work rate based on the patient’s body surface area (BSA) with linear increases every minute reaching a peak exercise after 10 min. Gas exchange was analyzed at rest, during exercise, and during recovery to determine measures of oxygen uptake (VO_2_) [20]. Since peak VO_2_ is influenced by age, sex, and body weight, the percent of predicted peak VO_2_ value (% predicted VO_2_) was used due to the wide age range in this study [21].

### Statistical analysis

The Student t-test or Mann-Whitney U test was used to compare two groups of continuous symmetric or non-symmetric variables, respectively, or Fisher exact for categorical variables, as appropriate. A normal approximation to binomial confidence interval was constructed for the percentage of patients with MPBF. Univariate association between normally distributed variables was estimated using the Pearson correlation coefficient. A stepwise multivariable linear regression modeling procedure with 0.1 as the significance level for entry and 0.05 as the significance level for retention in the model was constructed to determine independent predictors of % predicted VO_2_. A stepwise multivariable logistic regression model with 0.1 as the significance level for entry and 0.05 as the significance level for retention in the model was constructed to identify the independent factors associated with the presence or absence of MPBF. Continuous predictor variables were also categorized into tertiles to assess potential nonlinearity, but no nonlinear significant associations were found (data not shown). All *p*-values were two-tailed (where applicable) and differences and associations were considered significant when *p* < 0.05. Statistical analyses were performed using SPSS Statistics for Windows, Version 24.0 (International Business Machines, Armonk, New York, USA) and JMP®, Version 12 (SAS Institute Inc., Cary, North Carolina, USA).

## Results

There were 147 patients who met inclusion criteria with complete CMR PA blood flow data and CPET without interval intervention. Most patients had direct PA blood flow assessment in both PAs; there were 3 patients who had a PA stent requiring calculation using venae cavae flow measurements. The median age at CMR was 21.8 years [interquartile range (IQR) 16.5–30.6]. The median time between CMR and CPET was 2.8 months [IQR 0–13.8]. There was no significant difference in the time from CMR to CPET between patients with and without MPBF. Patient characteristics are summarized in Table 1 [4].

**Table 1:** Patient Demographic and Clinical Characteristics

	All Patients (*n* = 147)	Patients with MPBF (*n* = 53)	Patients without MPBF (*n* = 94)	*P* Value
Age at Fontan (yr)+	3.2 [2.2–6]	2.9 [2–4.8]	3.3 [2.3–7.6]	0.21
Age at CMR (yr)	21.8 [16.5–30.6]	20.7 [16.5–25]	23.2 [16.6–32.2]	0.20
Time since Fontan (yr)	17.2 [13.1–22.9]	16.5 [13.1–20.9]	17.9 [12.9–23.9]	0.23
Time between CMR and CPET (mos)	2.8 [0–13.8]	1 [0–14.5]	4.5 [0–11.5]	0.10
Body surface area at CMR (m^2^)	1.7 [1.5–1.9]	1.7 [1.5–1.8]	1.7 [1.5–2]	0.52
Cardiac Diagnosis				0.14
Tricuspid atresia	37 (25%)	14 (26%)	23 (24%)	
Double-inlet left ventricle	24 (16%)	7 (13%)	17 (18%)	
HLHS	25 (17%)	14 (26%)	11 (12%)	
Unbalanced AV canal	9 (6%)	4 (8%)	5 (5%)	
Double-outlet right ventricle	25 (17)	9 (17%)	16 (17%)	
Complex 2 ventricle	13 (9%)	2 (4%)	11 (12%)	
Hypoplastic TV/RV	6 (4%)	0 (0%)	6 (7%)	
Pulmonary atresia/IVS	2 (1%)	1 (2%)	1 (1%)	
Mitral atresia	6 (4%)	2 (4%)	4 (4%)	
Levocardia	122 (83%)	78 (83%)	44 (83%)	0.90
HLHS only	25 (17%)	14 (26%)	11 (12%)	0.04
Heterotaxy	17 (12%)	8 (16%)	9 (10%)	0.23
Genetic diagnosis	11 (7%)	5 (9%)	6 (6%)	0.45
Dominant ventricular morphology				0.68
Left ventricle	58 (40%)	19 (42%)	39 (42%)	
Right ventricle	49 (33%)	20 (32%)	29 (31%)	
2 ventricles	40 (27%)	14 (26%)	26 (27%)	
History of neonatal surgery	79 (54%)	34 (64%)	45 (48%)	0.06
History of Glenn operation	75 (51%)	33 (62%)	39 (41%)	0.02
History of Damus-Kaye-Stansel	42 (29%)	21 (40%)	21 (22%)	0.04
Bilateral Glenn operation	13 (8.8%)	5 (10%)	8 (10%)	0.50
Fontan Type				0.43
Lateral tunnel	98 (67%)	41 (77%)	57 (60%)	
RA-Pulmonary artery	31 (21%)	8 (15%)	23 (25%)	
Extracardiac	11 (8%)	3 (6%)	8 (11%)	
RA-RV Fontan	5 (3.4%)	1 (2%)	4 (4%)	
History of any PA intervention^a^	34 (23%)	20 (38%)	13 (14%)	0.004
History of PA intervention before Fontan	12 (8%)	10 (19%)	2 (2%)	0.001
History of PA intervention at Fontan	3 (2%)	1 (2%)	2 (2%)	0.70
History of PA intervention after Fontan	19 (13%)	10 (19%)	9 (10%)	0.09
Pulmonary vein stenosis^a^	5 (3%)	4 (7%)	1 (1%)	0.06

*Abbreviations*: *AV* atrioventricular, *CMR* cardiovascular magnetic resonance, *CPET* cardiopulmonary exercise test, *HLHS* hypoplastic left heart syndrome, *IVS* intact ventricular septum, *MPBF* maldistribution of pulmonary blood flow, *PA* pulmonary artery, *RA* right atrium, *RV* right ventricle, *TV* tricuspid valve

^a^Prior to CMR. + Data presented as median [interquartile range]

### Distribution of pulmonary blood flow

**Fig. 2:**
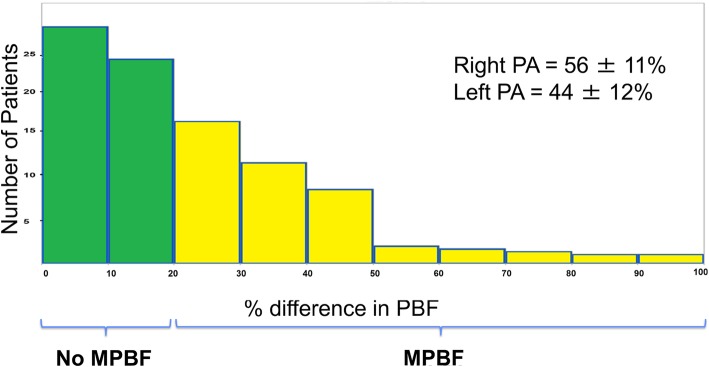
Percentage difference in branch PA blood flow between the two branch PAs. The mean branch PA flow is 56% to the right PA. Green denotes no MPBF (%difference < 20); yellow denotes MPBF (%difference ≥ 20). MPBF: Maldistribution of pulmonary blood flow. PA: Pulmonary artery. PBF: Pulmonary blood flow

**Fig. 3:**
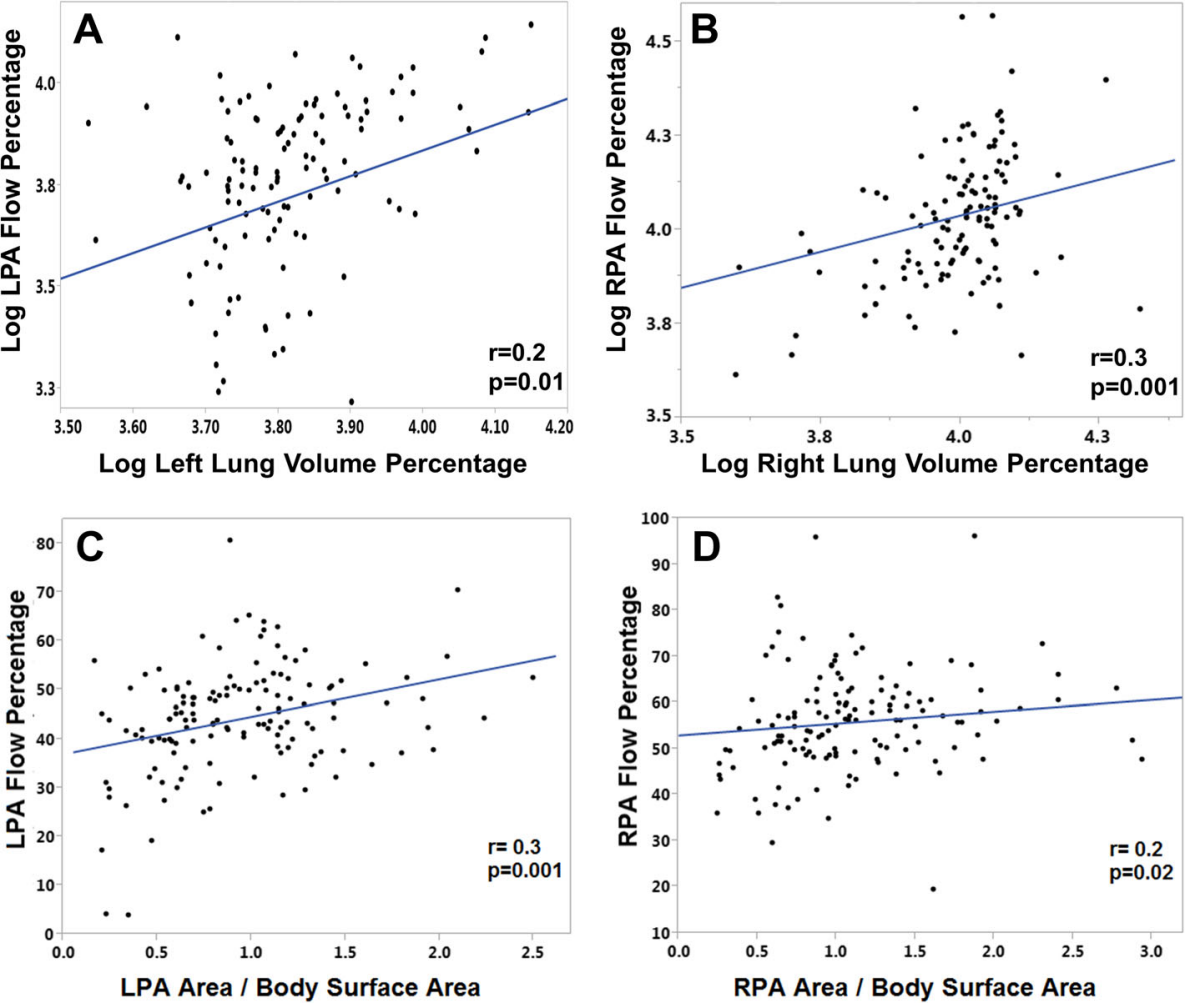
Association between branch PA flow percentage and lung volume percentage (**a**, **b**) and ipsilateral PA cross sectional area (**c**, **d**). *N* = 140. The estimate r denotes Pearson correlation coefficient. LPA: Left pulmonary artery. RPA: Right pulmonary artery. Log: Logarithmic transformation

MPBF was present in 53 patients (36%, 95% CI 29–45%). A histogram of differential PA blood flow is shown in Fig. 2. Total PA blood flow was 2.46 ± 0.6 L/m^2^. Average LPA blood flow was 1.07 ± 0.35 L/m^2^ which represented 44 ± 12% of total PA flow. Average RPA blood flow was 1.38 ± 0.44 L/m^2^ which represented 56 ± 11% of total PA flow. There was a weak correlation between branch PA flow percentage and ipsilateral PA cross-sectional area and less than 10% of the variability can be explained by cross sectional area (r^2^ < 0.1) (Fig. 3). Similarly there was a weak correlation between branch PA flow percentage and lung volume percentage (Fig. 3).

### MPBF and exercise capacity

**Fig. 4:**
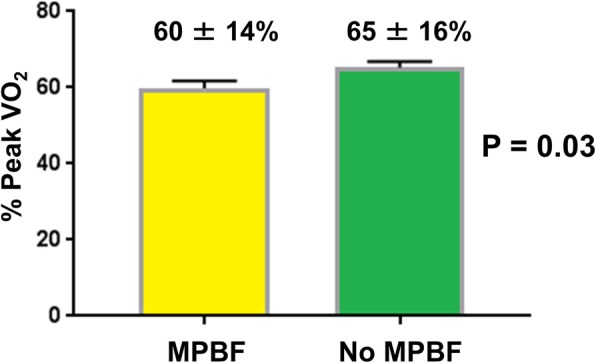
Patients with maldistribution of pulmonary blood flow (MPBF, *n* = 53; yellow) had lower mean % predicted peak VO_2_ compared to patients with no MPBF (*n* = 94, green). Error bars denote one standard deviation

The mean % predicted VO_2_ was 63 ± 16%. Patients with MPBF had lower % predicted VO_2_ compared to patients without MPBF (60 ± 14% versus 65 ± 16%; *p* = 0.04) (Fig. 4). Additional univariate associations of lower % predicted VO_2_ are shown in Table 2 and included longer time since Fontan, older age at the Fontan operation, atriopulmonary connection Fontan, heart failure symptoms, presence of a fenestration, and a higher ventricular mass-to-volume ratio. On multivariable analysis, only MPBF, time since Fontan, and ventricular mass-to-volume ratio were associated with a lower % predicted VO_2_ (Table 3).

**Table 2:** Univariate Associations with % Predicted VO_2_

Predictor	R or standardized β	Parameter Estimate ± SE	*P* value
Maldistribution of pulmonary blood flow (*n* = 147)	−0.17	−5.4 ± 2.6^a^	0.04
Mass-to-volume ratio (g/ml) (*n* = 140)	− 0.24	−19.38 ± 6.60^b^	0.004
Age at Fontan (year) (*n* = 139)	− 0.20	− 0.47 ± 0.19^b^	0.02
Old type Fontan (RA-PA or RA-RV conduit) (*n* = 146)	− 0.27	−9.2 ± 2.8^a^	0.001
CHF symptoms (*n* = 146)	− 0.21	−7.6 ± 2.9^a^	0.01
Atrial arrhythmia (*n* = 146)	− 0.29	−9.4 ± 2.6^a^	0.001
Fenestration (*n* = 139)	− 0.17	−1 ± 0.4^a^	0.04
Age at CMR (year) (*n* = 146)	− 0.36	− 0.55 ± 0.12^b^	0.001
Time since Fontan (year) (*n* = 139)	− 0.30	−0.67 ± 0.18^b^	0.001
Indexed end diastolic volume (ml/m^2^) (*n* = 145)	0.10	−0.03 ± 0.04^b^	0.90
Indexed end systolic volume (ml/m^2^) (*n* = 145)	−0.10	−0.07 ± 0.05^b^	0.16
Ejection fraction (%) (*n* = 145)	0.10	0.23 ± 0.14^b^	0.15
Indexed stroke volume (ml/m^2^) (*n* = 145)	0.01	0.01 ± 0.10^b^	0.94
Branch pulmonary artery symmetry index (*n* = 147)	0.04	2.52 ± 5.52^b^	0.64
Systemic left ventricle (*n* = 144)	0.01	−0.5 ± 3.0^a^	0.84
Heterotaxy (*n* = 147)	0.01	−3.3 ± 4.0^a^	0.41

*Abbreviations*: *PA* pulmonary artery, *RA* right atrium, *RV* right ventricle

^a^Mean difference ± SE for presence vs. absence of maldistribution of pulmonary blood flow

^b^Slope ± SE.SE = standard error

**Table 3:** Multivariable Model for % Predicted VO_2_ (*n* = 140)

Variable	Standardized β coefficient	Parameter Estimate ± SE	*P* value
Maldistribution of pulmonary blood flow	−0.20	−5.4 ± 2.6^a^	0.03
Time since Fontan (year)	−0.32	−0.67 ± 0.18^b^	0.002
Ventricular mass-to-volume ratio (g/ml)	−0.24	−19.38 ± 6.60^b^	0.02

^a^Mean difference ± SE for presence vs. absence of maldistribution of pulmonary blood flow

^b^Slope ± SE.SE = standard error

### MPBF and clinical outcomes

During a median follow-up period of 4.2 years [IQR 2.1–8.4] after the CMR, 14 (10%) patients died or were listed for heart transplant. Of the 10 patients who died, deaths were attributed to arrhythmias (*n* = 4), heart failure (*n* = 4), renal failure (*n* = 1), and protein losing enteropaty (*n* = 1). The follow-up period was similar among those with and without MPBF (Table 4). MPBF was not associated with death or listing for transplant (*p* = 0.60). MPBF was not associated with other comorbidities including atrial flutter, heart failure, protein-losing enteropathy, major thrombotic events, liver disease, or stroke (Table 4).

**Table 4:** Clinical Outcomes in Patients with and without MPBF

	All patients	With MPBF	Without MPBF	P value^*^
	(*n* = 147)	(*n* = 53)	(*n* = 94)	
Follow-up time post CMR (year)	4.2 [2.1–8.4]	4.1 [1.7–9.1]	4.4 [2.4–8.9]	0.59
Death	10 (7%)	4 (8%)	6 (6%)	0.70
Death / listing for transplantation	14 (10%)	6 (11%)	8 (9%)	0.60
Liver disease	50 (34%)	16 (30%)	34 (36%)	0.58
Heart failure	35 (24%)	15 (29%)	20 (22%)	0.42
Thrombus	28 (19%)	12 (22%)	16 (17%)	0.50
Seizures	16 (11%)	6 (11%)	10 (11%)	0.90
Stroke	26 (18%)	11 (21%)	15 (17)	0.50
Protein-losing enteropathy	5 (3%)	1 (2%)	4 (4%)	0.40
Atrial flutter post-Fontan	32 (22%)	11 (21%)	21 (22%)	0.90

*Abbreviations*: *MPBF* maldistribution of pulmonary blood flow

^*^Fisher exact test *p*-value for all variables except follow-up time (Mann-Whitney U Test)

### Parameters associated with MPBF

On univariate analysis, a surgical history of a Damus-Kaye-Stansel anastomosis, having a cardiac diagnosis of HLHS, prior Glenn procedure, and history of prior PA intervention were associated with MPBF (Table 1). The CMR parameters and their associations with MPBF are shown in Table 5. On univariate analysis, MPBF was associated with larger ventricular volumes, increased aortopulmonary collateral flow, larger ascending aorta and aortic root cross-sectional areas, PA compression by the ascending aorta or aortic root, PASI, and larger lung volume discrepancies. On multivariable logistic regression analysis, only PA compression by the ascending aorta or aortic root was associated with MPBF (OR = 6.5, 95% confidence interval 5.6–7.4, *p* < 0.001). Branch PA compression was seen in 32 patients (21%), and involved the LPA in 30 patients and the RPA in 2 patients.

**Table 5:** CMR Parameter Associations with MPBF

	All patients (*n* = 147)	With MPBF (*n* = 53)	Without MPBF (*n* = 94)	*P* value
Ventricular end-diastolic volume (ml/BSA^1.3^)	98 ± 29	104 ± 35	94 ± 25	0.05
Ventricular end-systolic volume (ml/BSA^1.3^)	49 ± 25	54 ± 26	46 ± 19	0.04
Aortopulmonary collateral flow (%)	12 [3–23]	16 [4–27]	10 [3–20]	0.02
Ascending aorta area (cm^2^)	4.9 ± 2.7	5.8 ± 3.7	4.4 ± 1.9	0.001
Total aortic root area (cm^2^)	6.9 ± 1.9	5.6 ± 1.7	5.1 ± 1.8	0.03
Pulmonary artery symmetry index (%)	65 ± 24	59 ± 25	68 ± 23	0.02
Pulmonary artery compression by the ascending aorta or aortic root	32 (22%)	21 (39%)	11 (12%)	0.001
Lung volume percentage difference (%)	13 ± 9	15 ± 7	12 ± 9	0.04
Indexed ventricular stroke volume (ml/m^2^)	57 ± 11	60 ± 13	56 ± 12	0.55
Ventricular mass-to-volume ratio (gram/ml)	0.55 ± 0.19	0.53 ± 0.20	0.58 ± 0.18	0.13
Indexed Ventricular mass (g/m^2^)	61.8 ± 19	62.0 ± 21	61.7 ± 18	0.77

Abbreviations: MPBF: maldistribution of pulmonary blood flow; BSA: body surface area

## Discussion

This study evaluated the PA blood flow distribution in 147 patients with a Fontan circulation. Patients with MPBF, defined as a difference between LPA and RPA blood flow of > 20%, had lower exercise capacity compared to patients without MPBF. Previous studies have shown that % predicted VO_2_ is an independent predictor of mortality in Fontan patients with a hazard ratio of 0.88 for each 1% increase in % predicted VO_2_ [22]. This would imply that our measured difference of 5% predicted VO_2_ is likely clinically significant. There were no other associations between MPBF and other clinical outcomes, including death or listing for transplantation. Patients with PA compression by a dilated ascending aorta or aortic root were the most likely to have MPBF.

Previous investigators have used CMR to evaluate PA blood flow distribution in normal subjects and patients with a Fontan circulation [4]. In individuals without congenital heart disease, 55% of PA blood flow is through the RPA and 45% through the LPA. This difference has been attributed to the smaller left lung volume due to the heart being in the left side of the chest [23]. In Fontan patients, Whitehead et al. demonstrated that, on average, RPA flow is 55% of the total PA flow. Likewise, our study showed similar flow to the RPA (56% of total PA blood flow) [24]. Both our study and previous studies revealed wide variations in PA blood flow distribution in Fontan patients. Whitehead et al. showed that the prevalence of MPBF is about 45%; however, the study was not designed to look at the associations with clinical outcomes or exercise capacity [4]. In our cohort, MPBF was common, seen in 36% of patients. The variability of PA blood flow distribution in our study is likely multifactorial and can be only partially explained by lung volume discrepancy as there was only a weak association with lung volumes.

Many variables were associated with MPBF in univariate analysis. On multivariable analysis, only branch PA compression by the ascending aorta or the aortic root had a significant association with MPBF. Previous studies showed that severe aortic root dilation or ascending aortic dilation is seen commonly in patients with Fontan circulation and was associated with aortic regurgitation [25]. Our study suggests that another adverse effect of aortic dilation is PA compression which can lead to MPBF, especially in patients with a left aortic arch leading to LPA compression. PA compression by the aortic root and the ascending aorta was recognized as a problem after the Stage I palliation that can lead to long-term PA hypoplasia in previous studies [26–28]. This resulted in multiple modifications of the surgical technique including changing the direction of the Blalock-Taussig shunt leftward into the retroaortic PA to avoid development of LPA stenosis and using more ring-enforced RV-PA conduits in addition to patch augmentation of the LPA [26–28].

Our study as with others found significant exercise impairment in Fontan patients (mean % predicted VO_2_ of 63%) [22, 29]. Previously reported determinants of exercise capacity include the inability to increase stroke volume at peak exercise, chronotropic impairment, diastolic dysfunction, and power loss in the Fontan circulation [30, 31]. Non-cardiac factors including age, muscle mass and conditioning are also important determinants [30, 31]. In addition to confirming the independent association of time since Fontan and ventricular mass-to-volume ratio with exercise capacity, our study introduces MPBF as another factor that adversely affects exercise capacity in the Fontan population.

We found a weak correlation between ipsilateral PA blood flow and PA size. These data would suggest that reliance solely on PA size (either by echocardiography or angiography) might not correlate well with differential PA blood flow. Direct PA blood flow assessment by CMR should be preferred.

MPBF may result in ventilation perfusion mismatch and less efficient gas exchange within the lungs [32]. MPBF, especially in the case of branch PA stenosis or compression, may also result in elevated Fontan baffle pressures which could lead to an increase in veno-venous collaterals to the pulmonary veins, which can cause systemic desaturation [33]. Fontan patients with severe unilateral branch PA stenosis have been noted to have significantly lower saturations compared to patients without stenosis [33]. Lower oxygen saturation has also been associated with lower exercise capacity in patients with congenital heart disease [4, 34, 35]. Finally, MPBF may also be associated with significant power loss in the Fontan circuit. Previous elegant work using computational flow dynamics showed that power loss correlates to the minimum cross sectional PA area in Fontan patients [36]. MPBF may be a surrogate for the potential power loss in the Fontan circuit [37]. It is also plausible that during exercise, the differences in PA blood flow may be magnified. This may explain why a small measured effect in MPBF at rest has significant impact on functional exercise capacity. Future real time CMR studies during exercise may increase our understanding of the relationships between MPBF, power loss, and exercise capacity.

### Limitations

This is a single-center CMR study which may limit the generalizability of these results to all Fontan patients. In particular, patients with pacemakers and defibrillators could not be evaluated; these devices are used in 13% of patients with Fontan circulation [37]. Sicker patients and patients with symptoms could be over-represented in this study, as these patients are more likely to be evaluated by CMR [38]. Also, 78 patients were excluded due to the incomplete flow data. Many of these patients had branch PA stents and may have had severe MPBF. Finally, this study included only patients with a maximal CPET, which limited the sample size and may have caused selection bias toward patients with higher functional status.

## Conclusion

In patients after the Fontan operation, MPBF was common, seen in more than one third of patients. Lower exercise capacity was independently associated with MPBF, longer time since Fontan, and increased ventricular mass-to-volume ratio. Patients with PA compression by the aortic root or the ascending aorta were more likely to have MPBF. This study identifies MPBF as an important risk factor and as a potential target for therapeutic interventions in this fragile patient population.

## Abbreviations

BSA: Body surface area
bSSFP: Balanced steady state free precession
CMR: Cardiovascular magnetic resonance
CPET: Cardiopulmonary exercise test
HLHS: Hypoplastic left heart syndrome
LPA: Left pulmonary artery
MPBF: Maldistribution of pulmonary blood flow
PA: Pulmonary artery
PASI: Pulmonary artery symmetry index
RPA: Right pulmonary artery
VO_2_: Oxygen update
